# A single session of low-volume high-intensity interval and moderate-intensity continuous exercise elicits a transient reduction in ghrelin levels, but not in post-exercise energy intake in obese men

**DOI:** 10.20945/2359-3997000000308

**Published:** 2020-11-09

**Authors:** Victor Araújo Ferreira Matos, Daniel Costa de Souza, Rodrigo Alberto Vieira Browne, Victor Oliveira Albuquerque dos Santos, Ítalo Freire Medeiros, Paulo Ricardo Porfírio do Nascimento, Eduardo Caldas Costa, Ana Paula Trussardi Fayh

**Affiliations:** 1 Universidade Federal do Rio Grande do Norte Programa de Pós-Graduação em Educação Física Natal RN Brasil Programa de Pós-Graduação em Educação Física, Universidade Federal do Rio Grande do Norte, Natal, RN, Brasil; 2 Universidade Federal do Rio Grande do Norte Programa de Pós-Graduação em Ciências da Saúde Natal RN Brasil Programa de Pós-Graduação em Ciências da Saúde, Universidade Federal do Rio Grande do Norte, Natal, RN, Brasil; 3 Universidade Federal do Rio Grande do Norte Departamento de Nutrição Natal RN Brasil Departamento de Nutrição, Universidade Federal do Rio Grande do Norte, Natal, RN, Brasil; 4 Universidade Federal do Rio Grande do Norte Instituto de Medicina Tropical Natal RN Brasil Instituto de Medicina Tropical, Universidade Federal do Rio Grande do Norte, Natal, RN, Brasil

**Keywords:** High-intensity interval training, appetite-regulating hormones, energy intake, hunger, obesity

## Abstract

**Objective:**

This study investigated the acute effects of high-intensity interval (HIIE) and moderate-intensity continuous (MICE) exercise on ghrelin levels in obese men.

**Subjects and methods:**

A total of 10 obese men (age 27.6 ± 1.8 years, body mass index 35.4 ± 4.5 kg/m², body fat 39.9 ± 2.1%) performed two exercise sessions in a randomized order: HIIE (10 × 1 min intervals at 90% of the maximal heart rate [HR_max_] interspersed by 1 min of active recovery) and MICE (20 min at 70% of the HR_max_). Ghrelin levels were assessed pre-, post- and 1h post-exercise, and energy intake was assessed 1h post-exercise through an *ad libitum* meal.

**Results:**

HIIE and MICE showed a trend to decrease ghrelin levels immediately post-exercise (-14.1 ± 21.6% and −9.6 ± 23.8%, respectively, p = 0.07) and decreased 1h post-exercise (-12.7 ± 31.8% and −13.8 ± 21.7%, respectively, p < 0.05). No changes were observed for post-exercise energy intake (p > 0.05). There was a positive correlation between the change in ghrelin levels and post-exercise energy intake only for HIIE (r = 0.63, p = 0.05).

**Conclusion:**

In summary, a single session of HIIE and MICE elicits a reduction on ghrelin levels without changing post-exercise energy intake in obese men.

## INTRODUCTION

Obesity has increased rapidly in recent decades and became a major public health concern given its association with chronic diseases, including hypertension, type 2 diabetes, and cancer (1). Obesity is a multifactorial disease characterized by an excessive fat accumulation resulting from the chronic disruption between energy intake and energy expenditure, involving genetic, environmental and lifestyle aspects (2,3).

Physical exercise leads to a negative energy balance and a cascade of central and peripheral compensatory adaptive mechanisms (4), playing a role in weight management and improvements in metabolic dysfunctions associated to obesity (2,5). In addition, physiological control of energy intake is an important aspect which may affect weight loss (6,7). These responses are mediated by complex interactions between key brain regions involved in energy homeostasis and circulating appetite-regulating hormones resulting in hunger and satiety episodes (8). In this context, ghrelin is considered a key appetite parameter given its concentration is associated with increased hunger and energy intake (7,9). This gastrointestinal hormone is the only known gut-derived peptide, is released from endocrine cells in the gastric mucosa, and plays an orexigenic role (10).

Previous studies have suggested an interesting aerobic exercise effect promoting a transient reduction in appetite-related parameters, including ghrelin levels in healthy (11-13) and overweight individuals (14-16). This response seems to be associated with the exercise intensity and its changes in energy substrates (i.e. increased blood lactate and glucose) (9,17,18). High-intensity interval exercise (HIIE) has emerged as a promising approach for treating obesity given its significant improvements to obesity-related comorbidities and reduced body fat (19,20); however, its effects on appetite related parameters and its relation with ghrelin and energy intake is not well known in obese individuals. To the best of our knowledge, only Sim and cols. (15) found reduced ghrelin levels following a single HIIE and moderate-intensity continuous exercise (MICE) sessions in overweight men, but a significant decrease in energy intake was only found after HIIE. On the other hand, Martins and cols. (16) observed a significant decrease in ghrelin levels after brief HIIE and MICE sessions, with no difference in subsequent energy intake for both conditions. A recent meta-analysis suggests that there is a moderate reduction in ghrelin levels after an acute exercise session in overweight/obese individuals (21). However, there is a need for a *head-to-head* comparison between the effects of HIIE and MICE on ghrelin levels and subsequent energy intake in obese individuals.

Therefore, this study compared the acute effects of HIIE and MICE on ghrelin levels in obese men and post-exercise energy intake. It was hypothesized that the ghrelin levels and post-exercise energy intake following a single HIIE session would be lower than after a MICE session in obese men.

## SUBJECTS AND METHODS

### Study design and participants

This study is a randomized controlled crossover trial including active obese men (body mass index [BMI] of 30 to 39.9 kg/m^2^), aged between 22 and 41 years and not using any drugs. Active smokers and patients with gastrointestinal disorders, overt hypothyroidism, diabetes mellitus, hypertension, anemia, active infection or cancer were excluded. Participants were recruited from an invitation disclosed in university settings, e-mails and online social networks in the city of Natal, Brazil. The present study was conducted in accordance with the Declaration of Helsinki, and all procedures involving humans were approved by the Research Ethics Committee of Federal University of Rio Grande do Norte (Protocol No. 976.389/2015; CAAE 42441015.5.0000.5568). Written informed consent was obtained from all subjects. The trial was registered in the Brazilian clinical trials platform (ReBEC, No. RBR-62kr6f).

### Procedures

The volunteers first attended the laboratory at 8 a.m. after 12 h of overnight fasting and remained there for the next 3 h. The first visit was used for familiarization and baseline measures, anthropometric assessment, followed by a graded exercise test (RT250, Movement^®^, Pompeia, Brazil) to determine the maximal treadmill velocity (MTV) and maximum heart rate (HR_max_) in the experimental sessions to be performed. The volunteers then randomly underwent two experimental sessions on the following visits. Each session was interspaced by one week. Participants registered their food consumption 24 h before the first experimental session and were instructed to consume the same foods and to not exercise 24 h prior to each subsequent session.

### Body composition

Weight and height were measured by a digital scale (BC 553, Tanita^®^, Arlington Heights, IL, USA) and a portable stadiometer (Personal Caprice Portatil, Sanny^®^, São Bernardo do Campo, SP, Brazil), respectively. BMI was calculated and the nutritional status of each participant was classified (22). Body composition was evaluated by double-energy X-ray absorptiometry (GE, Medical Systems, Chicago, IL, USA). The participants were instructed to avoid diuretics and caffeinated beverages the day before the evaluation (23).

### Maximal graded exercise test

The participants performed a warm-up on a treadmill (RT250, Movement^®^, Pompeia, Brazil) at a speed of 2.0 km/h for three minutes. Next, they started the incremental test at a speed of 3.0 km/h and increments of 1.0 km/h every minute until voluntary exhaustion. The MTV was considered as the highest velocity sustained by a full stage of one minute (24-26). Heart rate (HR) was monitored during the test using a HR monitor (RS800CX, Polar^®^, Kempele, Finland) and recorded at the end of each minute. The highest HR value observed during the test was considered as the maximal heart rate (HR_max_). Rating of perceived exertion (RPE) was also monitored during the test and recorded at the end of each minute according to the Borg scale 6-20 (27). The test ended when at least one of the following criteria was reached: (i) HR ≥ 100% estimated for age; (ii) RPE > 18; or (iii) when participants voluntarily stopped (28).

### Experimental sessions

The participants attended the laboratory after nocturnal fasting to perform the experimental sessions. The participants consumed a standardized liquid meal (Mass Titanium^®^, Max Titanium, Matão, Brazil) 60 minutes prior to both exercise sessions. The commercial product was powdery, and it was reconstituted in water to provide 4.5 kcal x weight (kg) to each participant. According to the manufacturer, each 100 g of powder has 377 kcal (87.5% of carbohydrates, 11.2% of proteins and 1.3% of lipids). The offered meal met the nutritional recommendations for pre-exercise calorie amount and macronutrient distribution (29).

The MICE session consisted of 20 min performed continuously at 50% of the HR_reserve_ or 70% of the HR_max_, which is the exercise intensity recommended as moderate by guidelines (28). The participants performed a 3 min warm-up at 4 km/h before both exercise sessions, and a 2 min cool-down at the same speed after the exercise sessions. The HIIE session was performed in a 1:1 “effort-recovery”. The participants performed 10×1 min work bouts at 90% of their individual MTV reached on the maximal graded exercise test (∼90% of the HR_max_), interspersed by 1 min of active recovery at 30% of MTV (i.e. slow walking). This low-volume HIIE model was used in previous studies (25,26,30). HR was continuously recorded throughout the exercise sessions (Polar Electro^®^, Oy, Finland). In addition, whole-body RPE was assessed using the RPE 6-20 Borg scale (27) during the last 10 s of each minute during the exercise sessions.

### Ghrelin and lactate

Total ghrelin was assessed by enzyme immunoassay with specific kits (Sigma Aldrich^®^, St. Louis, MO, USA) in the experimental sessions at three time points: “pre” (pre-exercise), 1h after standardized meal; “post” (immediately post-exercise); and “post 1h” (1h post-exercise). Based on previous studies (15,16,31), 1h post-exercise is appropriate to identify the acute effects of exercise on ghrelin levels. A trained professional withdrew 10 mL of blood from a vein in the antecubital region. The blood was subsequently centrifuged for 15 min at 3600 revolutions per minute. The serum was separated into 200 microliter aliquots and stored at −80 ºC for further analysis. Blood lactate was assessed “pre” (pre-exercise, 1h after standardized meal) and “post” (immediately post-exercise). The tip of the individual's finger was sanitized with a 70% alcohol solution and then pierced with a disposable lancet immediately after the HIIE and MICE sessions. Approximately 25 μL of blood was collected and analyzed on a specific portable monitor (Accutrend Plus^®^, Roche, Switzerland). Blood lactate was measured 2 minutes following the cool-down in all participants.

### Ad libitum energy intake

*Ad libitum* energy intake was measured through a meal offered one hour after the experimental sessions. The meal was offered in a reserved room as a “buffet” and participants were invited to eat “until they felt comfortably satisfied”. The following food options were part of this buffet: apples, bananas, toast, natural yogurt, potato chips, chocolate, fruit juice, boiled eggs, jam and butter. The food was weighed before and after consumption and dietary intake was performed with food analysis software (DietwinProfissional^®^ version 2016, Porto Alegre, Brazil). The selected foods presented in the meal were chosen according to the Brazilian population's food guide (32), which establishes guidelines for a healthy and adequate diet considering social, economic and cultural aspects of each region of Brazil.

### Statistical analysis

Data normality was verified by Shapiro-Wilk test, asymmetry and kurtosis (z-score). A paired t-test was used to compare the average of HR, RPE, lactate and ad libitum energy intake between the experimental conditions. Two-way repeated measures analysis of variance followed by Bonferroni's post hoc were used to verify the interaction effect of condition by time and main effects for ghrelin levels. The effect size of the variances was calculated by the partial squared eta (partial η^2^). Pearson's correlation coefficient was used to examine the relationship between relative change in ghrelin levels and energy intake values in each experimental condition. The significance level was adopted at p ≤ 0.05. The data were presented in mean ± standard deviation. All statistical procedures were performed using SPSS for Win v.25.0 (Statistical Package for Social Sciences, Chicago, IL, USA).

## RESULTS

Table 1 shows the characteristics of the participants. As expected, the results of BMI and body composition are compatible with the diagnosis of obesity.

**Table 1:** Characteristics of the participants (n = 10)

	Mean ± SD
Age (years)	27.6 ± 1.8
Weight (kg)	109.4 ± 18.3
Height (cm)	180 ± 8
Body mass index (kg/m²)	35.4 ± 4.5
Fat-free mass (kg)	65.4 ± 10.9
Body fat (%)	39.9 ± 2.1
**Habitual food intake**	
Energy intake (kcal)	2888 ± 656
Carbohydrates (%)	56.3 ± 5.6
Carbohydrates (g/kg/day)	3.8 ± 0.8
Protein (%)	15.6 ± 2.5
Protein (g/kg/day)	1.0 ± 0.1
Lipids (%)	28.1 ± 4.5
Lipids (g/kg/day)	0.9 ± 0.2

### Experimental sessions

The mean intensity of the HIIE session including work bouts and rest in intervals was higher than the MICE session (74 ± 8% *vs.* 52 ± 5% of the HR_reserve_ or 84 ± 5% *vs.* 71 ± 5% of the HR_max_, p < 0.01). The mean RPE responses to different exercise were higher in the HIIE session compared to the MICE session (13.6 ± 1.6 *vs.* 11.8 ± 1.1, p < 0.01). There was an increase in the blood lactate levels following both exercise sessions (HIIE: 3.3 ± 0.6 *vs.* 12.5 ± 2.5 mmol/L, p < 0.01; MICE: 3.1 ± 1.3 *vs.* 4.7 ± 1.6 mmol/L, p < 0.05). Furthermore, post-exercise blood lactate was higher in the HIIE sessions compared to the MICE session (p < 0.01).

### Ghrelin

**Figure 1:**
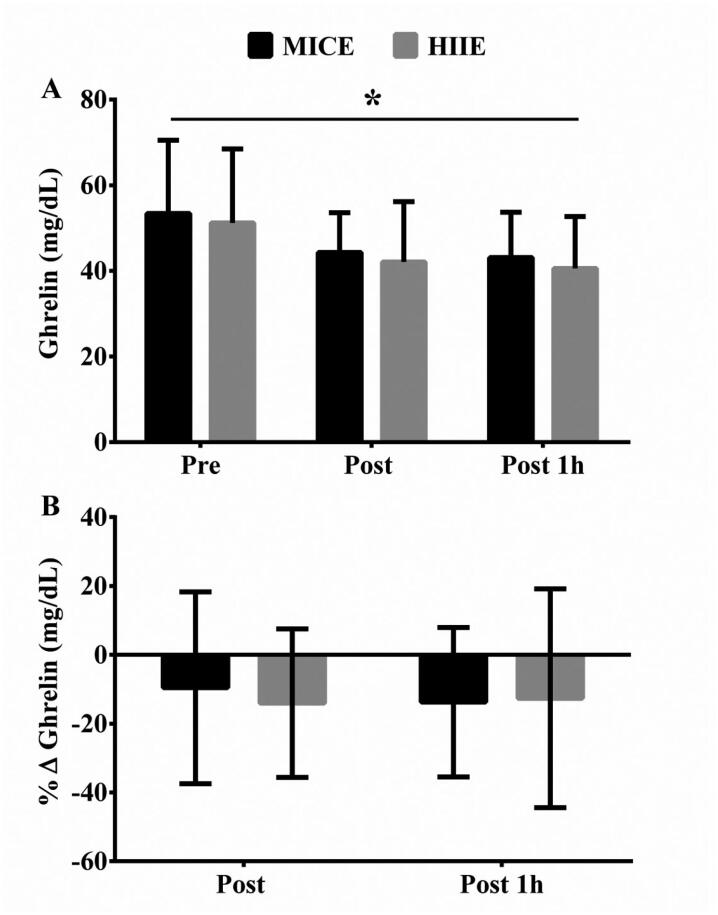
Ghrelin levels pre-exercise, immediately post-exercise and 1h post-exercise sessions.

HIIE: high-intensity interval exercise; MICE: moderate-intensity continuous exercise.

Figure 1 shows the ghrelin levels up to 1h post-exercise sessions. No significant interaction time by condition was observed, F (2, 18) = 0.00, p = 1.00, partial η^2^ = 0.00, power = 0.05. However, a significant main effect of time was observed, F (2, 18) = 7.75, p = 0.004, partial η^2^ = 0.46, power = 0.91. Bonferroni's post hoc analysis revealed that the ghrelin levels showed a trend to decrease immediately post-exercise (p = 0.07) and decreased 1-h post-exercise in both experimental conditions (p < 0.05, Figure 1A). Figure 1B shows the values of relative change in ghrelin levels. The MICE session showed a reduction of −9.6 ± 23.8 % and −13.8 ± 21.7% immediately and 1h post-exercise, respectively. HIIE showed a reduction of −14.1 ± 21.6% and −12.7 ± 31.8% immediately and one-hour after exercise, respectively.

Panel A shows the absolute values of ghrelin levels.

Panel B shows the delta of change (post – pre-exercise values) of ghrelin levels.

Data are reported as means ± standard deviation.

*Main effect of time for both exercise sessions (p < 0.05).

### *Ad libitum* post-exercise energy intake

**Figure 2:**
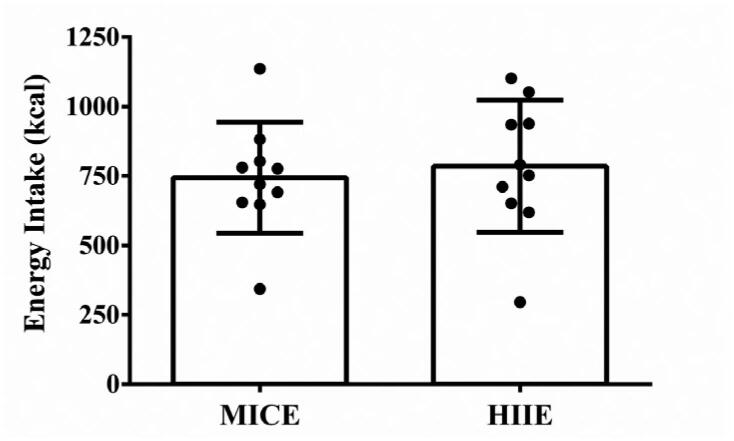
Energy intake in the post-exercise *ad libitum* meal.

HIIE: high-intensity interval exercise; MICE: moderate-intensity continuous exercise.

Figure 2 shows the *ad libitum* energy intake following the experimental conditions. There was no statistically significant difference between the MICE and HIIE sessions for post-exercise energy intake, t (9) = −0.987, p = 0.349. Similarly, there was no statistically significant difference for carbohydrates (88.3 ± 29.5 g *vs.* 95.4 ± 30.8 g; t (9) = 1.15, p = 0.280), proteins (25.6 ± 7.3 g *vs.* 95.4 ± 30.8 g; t (9) = 0.023, p = 0.982) or lipids (32.1 ± 11.3 g *vs.* 33.4 ± 11.5 g; t (9) = 0.546, p = 0.598).

Data are reported as means ± standard deviation and individual values (•).

### Correlation between ghrelin level and energy intake

**Figure 3:**
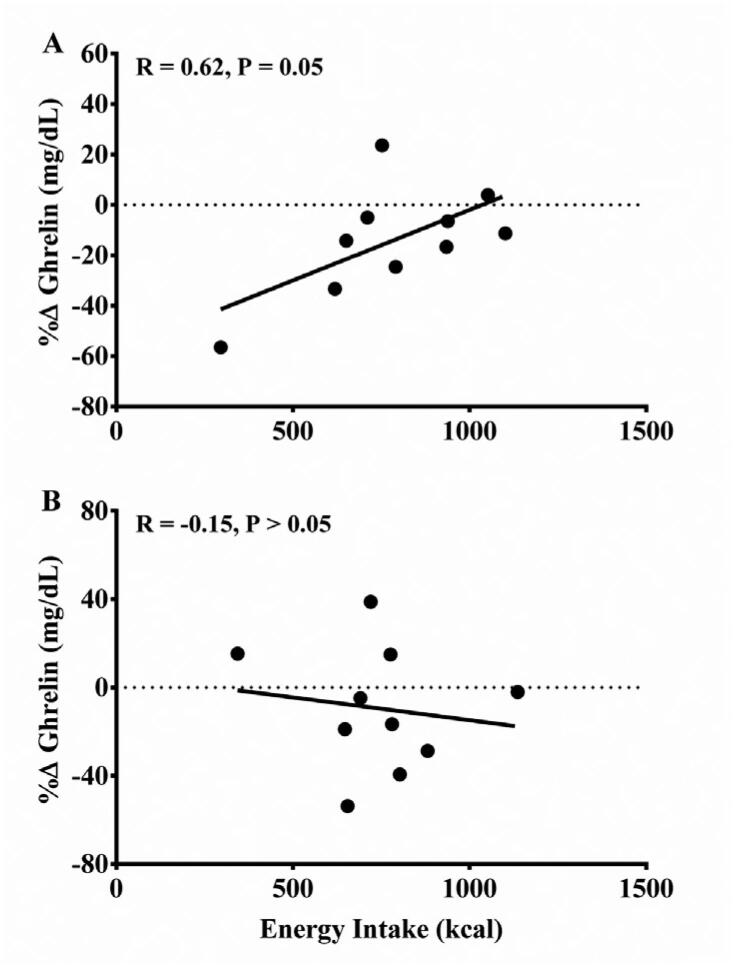
Correlation between the delta of change (post – pre-exercise values) of ghrelin levels and energy intake in the post-exercise *ad libitum* meal.

Figure 3 shows the correlation between relative change in ghrelin and ad libitum energy intake values following the experimental conditions. There was a positive correlation for HIIE (r = 0.62, p = 0.05). Despite this, there was no correlation for MICE (r = −0.15, p > 0.05).

Panel A refers to the high-intensity interval exercise session.

Panel B refers to the moderate-intensity continuous exercise session.

## DISCUSSION

The main findings of the present study were: i) the MICE and HIIE sessions elicited a significant reduction in the ghrelin levels up to 1h post-exercise; ii) there was a positive correlation between the change of ghrelin levels and post-exercise energy intake only for the HIIE session; iii) post-exercise energy intake was not different between the HIIE and MICE sessions. Therefore, the initial hypothesis that a greater suppression of the appetite in obese men occurs in the HIIE session compared to MICE was not confirmed.

A low-volume MICE and HIIE session elicited a reduction in the ghrelin levels up to 1h post-exercise. This finding is in agreement with previous studies that have included normal weight (12,33) and overweight individuals (15,16) submitted to different exercise intensity protocols. The current literature suggests that reduced ghrelin levels during exercise is associated with an increased blood flow to muscle tissue, resulting in a lower activation of ghrelin P1/D1 producing cell sin the stomach (9,31), which may explain our results. Importantly, no differences in the ghrelin levels were found between the MICE and HIICE sessions. Different from our results, previous studies have demonstrated a greater decrease of ghrelin levels following “all-out” sprint interval exercise (15,34), which is different from the protocol used in the present study that involved interval bouts at a vigorous intensity. Thus, it is reasonable to think that “all-out” interval exercise protocols may elicit a dissimilar response on post-exercise ghrelin levels compared to vigorous-intensity interval exercise. In addition, “low doses” of MICE and HIIE, as performed in the present study, may similarly decrease post-exercise ghrelin levels in obese men.

Acylated ghrelin is a peripheral hormone that is well known to stimulate hunger and food intake (31) which represents ∼10%-20% of total ghrelin (10). It has been extensively speculated that exercise intensity is a factor which may influence exercise-induced suppression of this peptide (9,12,15,31), and despite this hormone not being evaluated in the present study, it is possible that “low doses” of MICE and HIIE should also suppress acylated ghrelin in an obese population. However, future investigations are necessary to elucidate these effects of different exercise intensity protocols on acylated ghrelin response in obese subjects.

In addition, no significant differences were found in the energy intake and macronutrients between the MICE and HIIE sessions. Similarly, Martins and cols. (16) did not find differences between “all-out” interval exercise (8 s “all-out” bouts interspersed by 12 s at low-intensity) and MICE (70% of the HR_max_) on post-exercise energy intake. On the other hand, Sim and cols. (15) observed a decrease in post-exercise energy intake following two interval exercise protocols (60s at 100% of the VO_2peak_ interspersed by 240 s at 50% of the VO_2peak_; 15 s at 170% of the VO_2peak_ interspersed by 60 s at 32% of the VO_2peak_), but not following a MICE (60% of the VO_2peak_) session in inactive overweight men. Taken together, still little is known about the acute effects of MICE and HIIE on post-exercise energy intake, and the available studies have shown conflicting findings. Therefore, more data are needed for a better understanding of the potential role of “low doses” of aerobic exercise performed at different intensities (moderate *vs*. high) on post-exercise energy intake in obese populations.

Although no significant reduction in post-exercise energy intake was observed for the exercise conditions, a positive correlation between the change in ghrelin levels and the post-exercise energy intake was only found in the HIIE session. The increased blood flow directed to muscle tissue during higher physical exertion may reduce the activation of endocrine cells in the gastric mucosa, resulting in decreased ghrelin levels and subsequently a decrease in energy intake (9,15,33). In addition, we observed a higher post-exercise lactate in the HIIE compared to the MICE session (12.4 ± 2.5 *vs.* 4.7 ± 1.60 mmol/L). Therefore, a potential role of blood lactate should be considered given that it binds to ghrelin-producing cells during exercise and inhibits their secretory function (18,35). Finally, a previous study using similar exercise protocols in obese men found a transient suppression on hunger perception after HIIE, but not after MICE, which may contribute to a reduced post-exercise energy intake (30).

Regarding the strengths of the present study, there was a standardized meal before the exercise sessions in order to minimize individual changes in appetite. Furthermore, a post-exercise *ad libitum* energy intake measurement was implemented which enabled detecting food consumption as a “practical variable” together with biochemical variables (ghrelin and lactate levels). However, the present study has some limitations which should be mentioned. The exercise protocols were matched by time (20 min/session), but not by energy expenditure. Therefore, we do not rule out the possibility of there being bias related to higher calorie expenditure in the HIIE compared to the MICE session. Second, the absence of a control condition may have prevented a greater observation on basal ghrelin levels, although we did consider the pre-exercise values. Previous studies (13,15,16) have shown that both MICE and HIIE elicit a decrease in post-exercise *ad libitum* energy intake and ghrelin levels compared to a control condition without exercise. Thus, based on these studies (13,15,16) we have not included a control condition given that the main aim of the present study is *head-to-head* comparisons between MICE and HIIE protocols in obese men.

As a practical perspective, “low doses” of HICE and MICE might elicit reductions on ghrelin levels in obese men up to 1h post-exercise, which can contribute to facilitate a negative energy balance mediated by a transient appetite suppression. Future investigations on additional appetite biomarkers (PYY, CCK, insulin) and energy balance variables (respiratory exchange rate, energy expenditure during exercise) are important to elucidate such responses in obese populations. In summary, both low-volume HIIE and MICE elicit a transient reduction on ghrelin levels up to 1h post-exercise, without a difference in subsequent *ad libitum* post-exercise energy intake in obese men.

## References

[1] Cefalu WT, Bray GA, Home PD, Garvey WT, Klein S, Pi-Sunyer FX (2015). Advances in the Science, Treatment, and Prevention of the Disease of Obesity: Reflections From a Diabetes Care Editors’ Expert Forum. Diabetes Care.

[2] Ross R, Dagnone D, Jones PJ, Smith H, Paddags A, Hudson R (2000). Reduction in obesity and related comorbid conditions after diet-induced weight loss or exercise-induced weight loss in men. A randomized, controlled trial. Ann Intern Med.

[3] Heymsfield SB, Wadden TA (2017). Mechanisms, Pathophysiology, and Management of Obesity. N Engl J Med.

[4] MacLean PS, Blundell JE, Mennella JA, Batterham RL (2017). Biological control of appetite: A daunting complexity. Obesity (Silver Spring).

[5] Trussardi Fayh AP, Lopes AL, Fernandes PR, Reischak-Oliveira A, Friedman R (2013). Impact of weight loss with or without exercise on abdominal fat and insulin resistance in obese individuals: a randomised clinical trial. Br J Nutr.

[6] King NA, Caudwell P, Hopkins M, Byrne NM, Colley R, Hills AP (2007). Metabolic and Behavioral Compensatory Responses to Exercise Interventions: Barriers to Weight Loss. Obesity (Silver Spring).

[7] Hussain SS, Bloom SR (2013). The regulation of food intake by the gut-brain axis: implications for obesity. Int J Obes.

[8] Harrold JA, Dovey TM, Blundell JE, Halford JCG (2012). Neuropharmacology Invited review CNS regulation of appetite. Neuropharmacology.

[9] Hazell TJ, Islam H, Townsend LK, Schmale MS, Copeland JL (2016). Effects of exercise intensity on plasma concentrations of appetite-regulating hormones: Potential mechanisms. Appetite.

[10] Kojima M, Kenji K (2005). Ghrelin: Structure and Function. Physiol Rev.

[11] King NA, Burley VJ, Blundell JE (1994). Exercise-Induced Suppression of Appetite: Effects on Food Intake and Implications for Energy Balance. Eur J Clin Nutr.

[12] Broom DR, Stensel DJ, Bishop NC, Burns SF, Miyashita M (2007). Exercise-induced suppression of acylated ghrelin in humans. J Appl Physiol (1985).

[13] Panissa VLG, Julio UF, Hardt F, Kurashima C, Lira FS, Takito MY (2016). Effect of exercise intensity and mode on acute appetite control in men and women. Appl Physiol Nutr Metab.

[14] Matos VAF, Souza DC de, Browne RAV, Santos VOA dos, Costa EC, Fayh APT (2017). Acute effect of high-intensity interval exercise and moderate-intensity continuous exercise on appetite in overweight/obese males: a pilot study. Sport Sci Health.

[15] Sim AY, Wallman KE, Fairchild TJ, Guelfi KJ (2014). High-intensity intermittent exercise attenuates ad-libitum energy intake. Int J Obes (Lond).

[16] Martins C, Stensvold D, Finlayson G, Holst J, Wisloff U, Kulseng B (2015). Effect of moderate- and high-intensity acute exercise on appetite in obese individuals. Med Sci Sports Exerc.

[17] Peake JM, Tan SJ, Markworth JF, Broadbent JA, Skinner TL, Cameron-smith D (2014). Metabolic and hormonal responses to isoenergetic high-intensity interval exercise and continuous moderate-intensity exercise. Am J Physiol Endocrinol Metab.

[18] Islam H, Townsend LK, McKie GL, Medeiros PJ, Gurd BJ, Hazell TJ (2017). Potential involvement of lactate and interleukin-6 in the appetite-regulatory hormonal response to an acute exercise bout. J Appl Physiol (1985).

[19] Boutcher SH (2011). High-intensity intermittent exercise and fat loss. J Obes.

[20] Kessler HS, Sisson SB, Short KR (2012). The Potential for High-Intensity Interval Training to Reduce Cardiometabolic Disease Risk. Sports Med.

[21] Douglas JA, Deighton K, Atkinson JM, Sari-Sarraf V, Stensel DJ, Atkinson G (2016). Acute Exercise and Appetite-Regulating Hormones in Overweight and Obese Individuals : A Meta-Analysis. J Obes.

[22] (2000). Obesity: preventing and managing the global epidemic. Report of a WHO consultation. World Heal Organ Tech Rep Ser.

[23] Albanese CV, Diessel E, Genant HK (2003). Clinical applications of body composition measurements using DXA. J Clin Densitom.

[24] Frazão DT, de Farias LF, Dantas TC, Krinski K, Elsangedy HM, Prestes J (2016). Feeling of pleasure to high-intensity interval exercise is dependent of the number of work bouts and physical activity status. PLoS One.

[25] Fayh APT, Matos V, Souza DC, Santos VO, Marinho C, Serquiz A (2018). Effects of a single session of high-intensity interval exercise and moderate-intensity continuous exercise on biochemical cardiovascular risk factors in obese males. Sport Sci Health.

[26] de Souza DC, Matos VAF, Dos Santos VOA, Medeiros IF, Marinho CSR, Nascimento PRP (2018). Effects of High-Intensity Interval and Moderate-Intensity Continuous Exercise on Inflammatory, Leptin, IgA, and Lipid Peroxidation Responses in Obese Males. Front Physiol.

[27] Borg GA (1982). Psychophysical bases of perceived exertion. Med Sci Sports Exerc.

[28] Garber CE, Blissmer B, Deschenes MR, Franklin BA, Lamonte MJ, Lee IM (2011). Quantity and quality of exercise for developing and maintaining cardiorespiratory, musculoskeletal, and neuromotor fitness in apparently healthy adults: Guidance for prescribing exercise. Med Sci Sports Exerc.

[29] Thomas DT, Erdman KA, Burke LM (2016). American College of Sports Medicine Joint Position Statement. Nutrition and Athletic Performance. Med Sci Sport Exerc.

[30] Matos VAF, Souza DC, Santos VOA, Medeiros ÍF, Browne RAV, Nascimento PRP (2018). Acute effects of high-intensity interval and moderate-intensity continuous exercise on GLP-1, appetite and energy intake in obese men: A crossover trial. Nutrients.

[31] Schubert MM, Sabapathy S, Leveritt M, Desbrow B (2014). Acute exercise and hormones related to appetite regulation: a meta-analysis. Sports Med.

[32] Brazil. Ministry of Health (2014). Dietary Guidelines for the Brazilian Population.

[33] Deighton K, Karra E, Batterham RL, Stensel DJ (2013). Appetite, energy intake, and PYY3-36 responses to energy-matched continuous exercise and submaximal high-intensity exercise. Appl Physiol Nutr Metab.

[34] Holliday A, Blannin AK (2017). Very low volume sprint interval exercise suppresses subjective appetite, lowers acylated ghrelin, and elevates GLP-1 in overweight individuals: A pilot study. Nutrients.

[35] Engelstoft MS, Park WM, Sakata I, Kristensen LV, Husted AS, Osborne-Lawrence S (2013). Seven transmembrane G protein-coupled receptor repertoire of gastric ghrelin cells. Mol Metab.

